# Osteocalcin: A Potential Marker of Peripheral Arterial Stiffness in Hypertensive Patients

**DOI:** 10.3390/medicina60050835

**Published:** 2024-05-20

**Authors:** Yung-Hsuan Wang, Chien-Hao Hsiao, Ji-Hung Wang, Bang-Gee Hsu

**Affiliations:** 1 Division of Chest Medicine, Hualien Tzu Chi Hospital, Buddhist Tzu Chi Medical Foundation, Hualien 97004, Taiwan; 2 Division of Cardiology, Hualien Tzu Chi Hospital, Buddhist Tzu Chi Medical Foundation, Hualien 97004, Taiwan; allen80413@gmail.com; 3 School of Medicine, Tzu Chi University, Hualien 97004, Taiwan; 4 Division of Nephrology, Hualien Tzu Chi Hospital, Buddhist Tzu Chi Medical Foundation, Hualien 97004, Taiwan

**Keywords:** osteocalcin, hypertension, peripheral arterial stiffness, brachial–ankle pulse wave velocity, age

## Abstract

*Background and Objectives*: Brachial–ankle pulse wave velocity (baPWV) is an established independent risk factor for cardiovascular events, cardiovascular mortality, and all-cause mortality. Osteocalcin (OC) is recognized to be associated with vascular function. The present study assessed the correlation between serum OC levels and peripheral arterial stiffness (PAS) measured through baPWV in hypertensive patients. *Materials and Methods*: Fasting blood samples were collected from 120 hypertensive participants. The serum total OC levels were measured using a commercial enzyme-linked immunosorbent assay kit, whereas the baPWV device was used to detect PAS. The PAS group had left or right baPWV > 18.0 m/s. *Results*: Among the hypertensive patients, 24 (20.0%) were classified into the PAS group. The PAS group exhibited a significantly older age (*p* = 0.011), higher prevalence of diabetes (*p* = 0.010), systolic blood pressure (*p* = 0.019), levels of serum fasting glucose (*p* = 0.003), blood urea nitrogen (*p* = 0.024), creatinine (*p* = 0.004), C-reactive protein (*p* = 0.007), OC (*p* = 0.002), and lower estimated glomerular filtration rate (*p* = 0.004) than the non-PAS group. Age (odds ratio [OR]: 1.076, 95% CI: 1.004–1.153, *p* = 0.037) and serum OC level (OR: 1.797, 95% confidence interval (CI): 1.077–3.000, *p* = 0.025) were independent factors linked to PAS in hypertensive patients in the multivariate logistic regression analysis. *Conclusions*: Serum OC levels and older age are positively associated with PAS in hypertensive patients.

## 1. Introduction

Hypertension, with a high global prevalence, is a significant risk factor for cardiovascular disease (CVD) [1]. The common cardiovascular risk factors among hypertensive patients include age, diabetes mellitus (DM), albuminuria, atherosclerosis, dyslipidemia, hyperglycemia, smoking, and obesity [2,3]. Additionally, arterial stiffness is a significant predictor of cardiovascular mortality in hypertensive patients [4]. Increased arterial stiffness is associated with hypertension and organ damage [5].

Brachial–ankle pulse wave velocity (baPWV) is a method for measuring arterial stiffness. It is a non-invasive tool used to evaluate the stiffness of the arterial walls [6]. BaPWV is highly dependent on the muscle arteries of the lower limbs [7]. Elevated arterial stiffness in the lower extremities leads to the amplification and acceleration of wave reflections at the aortic level, increases in pulse pressure and afterload, and reduction in coronary artery perfusion [7]. An elevated baPWV value signifies elevated peripheral arterial stiffness (PAS), which is linked to an elevated risk of CVD and is also noted in CVD development in individuals with hypertension [8,9,10].

Osteocalcin (OC) is a bone-derived hormone that controls parasympathetic tone, muscle mass, brain development, glucose metabolism, and testosterone synthesis [11]. The OC activity depends on vitamin K and is associated with vascular calcification [12]. High serum OC levels are associated with arterial stiffness in patients with chronic kidney disease, as measured by the augmentation index normalized to a heart rate of 75 bpm (AIx75) [13]. In patients receiving peritoneal dialysis, elevated serum OC levels are also linked to arterial stiffness as determined by carotid-femoral pulse wave velocity [14]. Hence, exploring potential relationships between arterial stiffness and serum OC levels in hypertensive individuals is critical. We aimed to investigate the association between serum OC levels and PAS in hypertensive patients as measured by baPWV.

## 2. Materials and Methods

### 2.1. Patients

Between August 2020 and January 2021, we enrolled 120 hypertension patients from the cardiovascular outpatient department of a medical center located in Hualien, Taiwan. Acute myocardial infarction, heart failure, cancer, amputation, acute infection during blood collection, and refusal to provide informed consent for the study were among the exclusion criteria. The study was authorized by the Hualien Tzu Chi Hospital’s Research Ethics Committee, which is part of the Buddhist Tzu Chi Medical Foundation. Every research subject provided written informed permission.

Patients were diagnosed with DM if their fasting plasma glucose levels were ≥126 mg/dL or if they were taking hypoglycemic agents. Coronary artery disease (CAD) was defined as the presence of >50% stenosis in any segment based on the coronary angiography findings.

### 2.2. Anthropometric Analysis

A consistent operator conducted all anthropometric measurements, with participants dressed in light clothing and without shoes. Their body weight and height were documented to the closest 0.5 kg and 0.5 cm, respectively. The body mass index was computed by dividing the weight in kilograms by the square of the height in meters.

### 2.3. Biochemical Investigations

Participants gave about 5 mL of blood samples, which were centrifuged at 3000× *g* for 10 min after an 8–12-h fast. An autoanalyzer was used to measure the levels of serum blood urea nitrogen (BUN), creatinine, triglycerides (TG), total cholesterol, high-density lipoprotein cholesterol (HDL-C), low-density lipoprotein cholesterol (LDL-C), fasting glucose, total calcium, phosphorus, and C-reactive protein (CRP) (Siemens Advia 1800; Siemens Healthcare GmbH, Henkestr, Germany). The serum intact parathyroid hormone (iPTH) concentrations (Abcam, Cambridge, MA, USA) and serum total OC levels (eBioscience Inc., San Diego, CA, USA) were determined using commercially available enzyme-linked immunosorbent assays [14,15]. The Chronic Kidney Disease Epidemiology Collaboration equation was utilized to compute the estimated glomerular filtration rate (eGFR).

### 2.4. Systolic Blood Pressure, Diastolic Blood Pressure and baPWV Measurements

After blood sampling, the patients rested supine for 10 min. Trained staff measured the morning blood pressure (BP) using an upper arm automatic oscillometric device. Systolic BP (SBP) and diastolic BP (DBP) at the right brachial artery were recorded three times at 5-min intervals, and the average was calculated for analysis. Hypertension was defined as SBP of ≥140 mmHg and DBP of ≥90 mmHg, or as those who had used antihypertensive medication within the previous two weeks, in accordance with the Eighth Joint National Committee (JNC 8) standards for the prevalence survey. Four pneumatic cuffs connected to oscillometric and plethysmographic sensors encircled the two upper arms and ankles using a volume plethysmographic apparatus to measure the baPWV (VP-2000, Omron, Kyoto, Japan) [16]. The Moens–Korteweg equation in the field of biomechanics presented the connection between pulse wave velocity (PWV) and factors associated with blood vessels: PWV^2^ = E·h/r·p. E = elastic modulus, h = vessel wall thickness, r = vessel radius, and *p* = blood density. This equation states that the PWV can be increased by decreasing the functional/structural elasticity, increasing the vessel wall thickness, and/or decreasing the vessel diameter [6]. The Physiological Diagnosis Criteria for Vascular Failure Committee of Japan determined a baPWV of 18 m/s as a statistically appropriate cutoff point for patients with hypertension and those at high risk of CVD based on the results of their recursive partitioning study [6,17]. The patients with left or right baPWV values of >18.0 m/s were categorized as the PAS group.

### 2.5. Statistical Analysis

After determining whether the data were regularly distributed using the Kolmogorov–Smirnov test, the means ± standard deviation were shown and compared between patients using the Student’s independent *t*-test (two-tailed). The Mann–Whitney U test was used to compare non-normally distributed data, such as TG, fasting glucose, BUN, iPTH, and CRP levels, which were reported as medians and interquartile ranges. Logarithmic transformation was applied to non-normally distributed data to achieve normality for subsequent analysis. The chi-squared test was utilized to investigate categorical variables. Serum OC levels were compared to other variables using Spearman’s rank-order correlation coefficient. Multivariate logistic regression analysis was conducted for variables significantly correlated with PAS. After determining the impact of the OC level on the PAS, a receiver-operating curve (ROC) was created, and the area under the curve (AUC) was computed to calculate the power. IBM SPSS Version 19.0 (SPSS, Inc., Chicago, IL, USA) was used for all analyses, with a significance level of *p* < 0.05.

## 3. Results

The demographic, clinical, laboratory, and pharmaceutical profiles of patients with hypertension are shown in Table 1. Among them, DM affected 52 (43.3%), CAD affected 82 (68.3%), and PAS affected 24 (20.0%). In comparison to the control group, the PAS group was older (*p* = 0.011), had poorer eGFR (*p* = 0.004), higher serum fasting glucose (*p* = 0.003), increased serum BUN (*p* = 0.024), creatinine (*p* = 0.004), CRP (*p* = 0.007), and a greater number of DM patients (*p* = 0.010). There were no discernible variations between the two groups in terms of the distribution of sexes, the prevalence of concurrent CAD, or the rate of use of antihypertensive or anti-lipid drugs.

In the multivariate logistic regression analysis, which was adjusted for factors significantly correlated with PAS, including DM, age, SBP, fasting glucose, eGFR, CRP, and OC, we identified that serum OC levels (odds ratio [OR]: 1.797, 95% confidence interval (CI): 1.077–3.000, *p* = 0.025) and age (OR: 1.076, 95% CI: 1.004–1.153, *p* = 0.037) were found to be independently correlated with PAS in patients with hypertension (Table 2).

**Figure 1:**
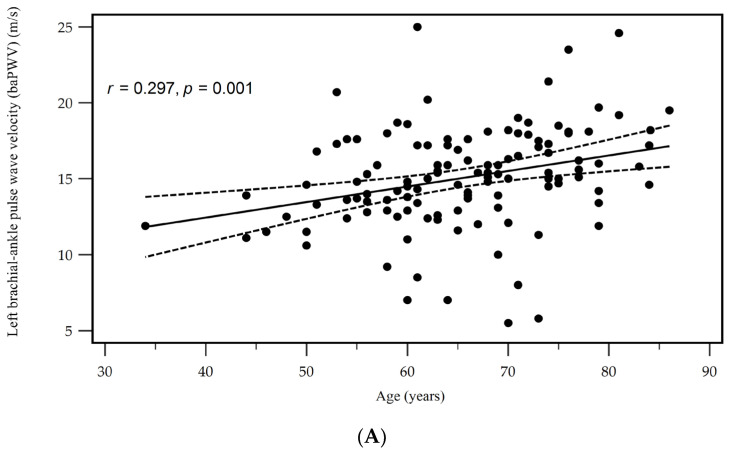

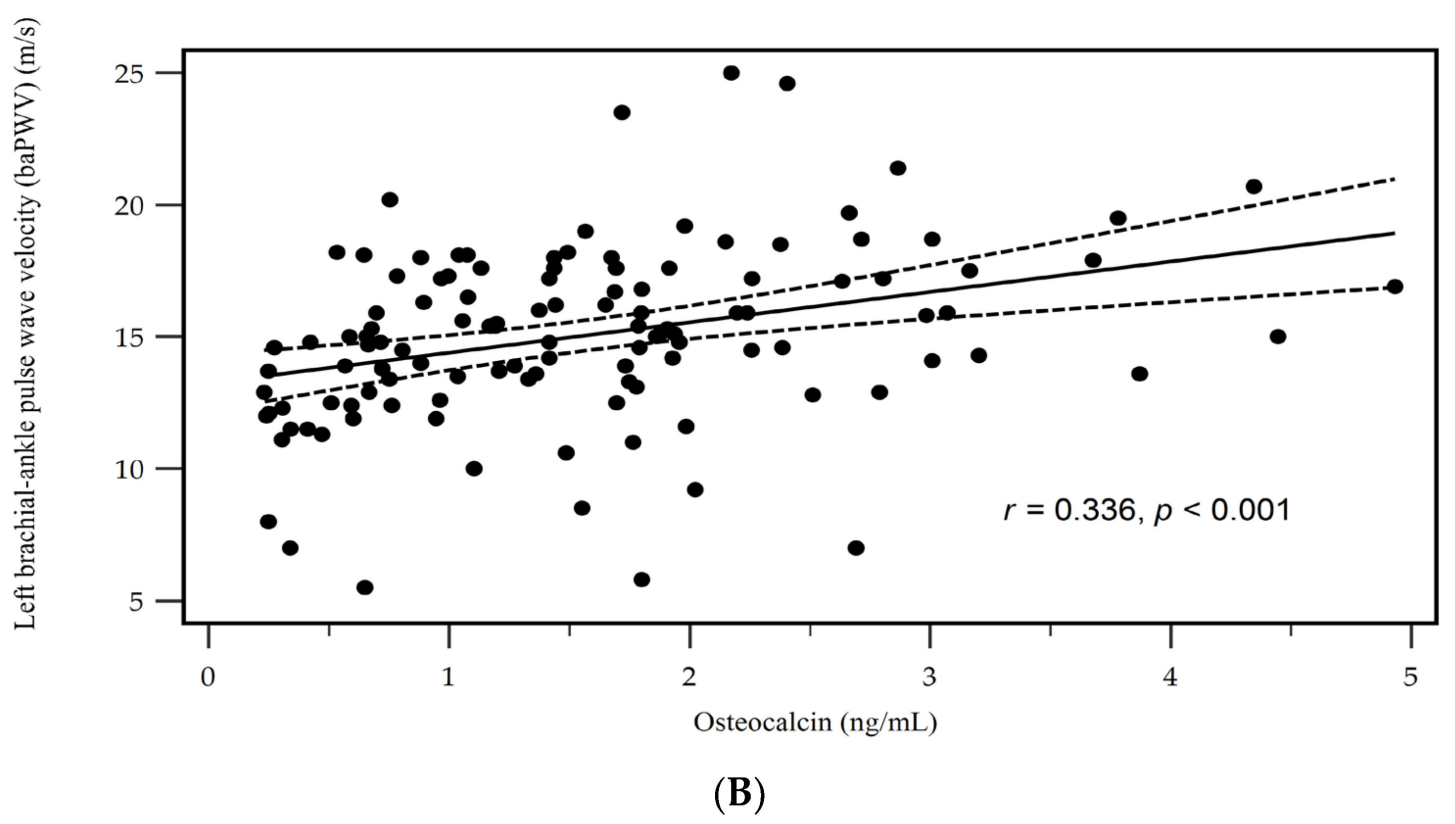
Scatter plots of age (**A**) and osteocalcin (**B**) levels with left brachial-ankle pulse wave velocity levels among hypertensive patients.

**Figure 2:**
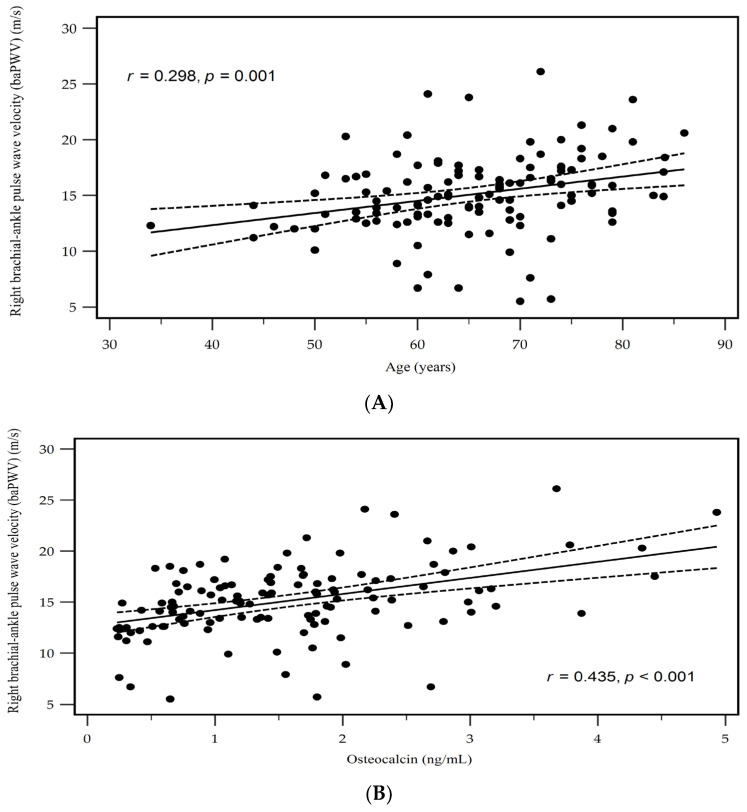
Scatter plots of age (**A**) and osteocalcin (**B**) levels with right brachial-ankle pulse wave velocity levels among hypertensive patients.

Table 3 illustrates the results of Spearman’s rank correlation coefficient test, which examined the association between the OC levels and clinical variables. Serum logarithmically transformed (log)-CRP (log-CRP) showed a positive correlation (*r* = 0.211, *p* = 0.021), whereas the eGFR exhibited a negative correlation (*r* = −0.208, *p* = 0.023) with the OC levels in patients with hypertension. Additionally, a positive association was found between the serum OC levels and either the left or right baPWV (*r* = 0.336, *p* < 0.001 and *r* = 0.435, *p* < 0.001, respectively) in hypertensive patients according to the Spearman’s rank correlation coefficient test. Two-dimensional scattered plots depicting the left and right baPWV values in a subgroup analysis based on age and OC levels among these hypertensive patients are presented in Figure 1A,B and Figure 2A,B, respectively.

**Figure 3:**
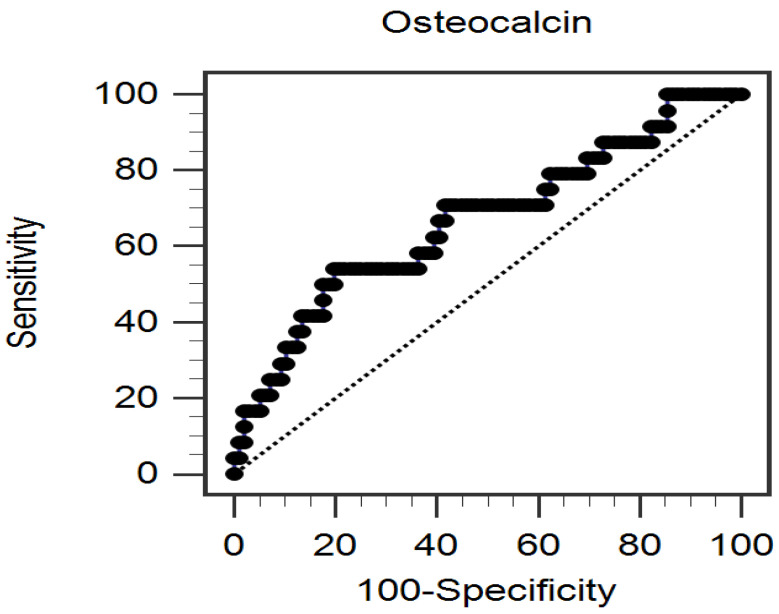
Peripheral arterial stiffness can be predicted by serum osteocalcin levels, as indicated by the area under the receiver operating characteristic curve.

The predictive value of serum OC levels for PAS occurrence was investigated through a ROC curve analysis, which resulted in an optimal cutoff value of 1.96 ng/mL by Youden’s J statistic method with 54.17% sensitivity, 80.21% specificity, 40.63% positive predictive value, and 87.50% negative predictive value. The AUC for the ROC curve to predict PAS by serum OC level was 0.668 (95% CI = 0.538–0.798, *p* = 0.011) (Figure 3).

## 4. Discussion

Our results indicated that older age and higher serum OC levels are associated with PAS in hypertensive patients; specifically, serum OC levels are positively associated with serum log-CRP levels and left or right baPWV values and negatively associated with eGFR in patients with hypertension.

Arterial stiffness involves complex processes, including changes in the extracellular matrix characterized by reduced elastin and increased collagen content [18]. Aging contributes to decreased arterial wall elasticity and compliance and influences arterial stiffness [19,20]. Arterial stiffness is reportedly higher in patients with DM than those without DM [20,21]. Diabetic *microvascular diseases*, including neuropathy, retinopathy, and nephropathy, are closely associated with arterial stiffness [21]. Additionally, a prospective cohort research found an independent relationship between arterial stiffness as measured by estimated pulse wave velocity (ePWV) and the all-cause and cause-specific mortality risks in DM patients [22]. This study noted a median of 115 months of follow-up in 5235 DM patients. After the adjusted Cox regression model, every 1 m/s increase in ePWV was associated with a 56% increase in the risk of all-cause mortality. Furthermore, a nonlinear correlation was found between all-cause mortality and ePWV. With the exception of accidents and deaths from renal diseases, the probability of the majority of cause-specific deaths increased from 53% to 102% for every 1 m/s increase in ePWV [22]. High BP was another risk factor linked to a faster increase in baPWV [6] in the STEP trial (Strategy of Blood Pressure Intervention in the Elderly Hypertensive Patients) of a cross-lagged panel model in the 5369 participants. In both the intensive treatment groups (SBP target, 110–130 mm Hg) and standard treatment groups (SBP target, 130–150 mm Hg), arterial stiffness measured by baPWV was a consistent predictor of SBP and made it more difficult to achieve target SBP, especially in the intensive therapy group [23]. Chronic renal failure is also a risk factor for arterial stiffness [6,20]. A cross-sectional study noted a non-linear association between baPWV and eGFR. When eGFR is <77.05 mL/min, a negative correlation between eGFR and baPWV was found [24]. Furthermore, in patients with CKD, a high baPWV was linked to the course of the CKD progression, the time it took to start dialysis or even death [25]. Inflammation has been linked to arterial stiffness because persistent inflammation contributes to arterial aging [20]. Even after accounting for potential confounders in 6572 consecutive participants, higher baPWV and high-sensitivity CRP (hs-CRP) levels were linked to cardiovascular death, acute myocardial infarction, coronary revascularization, and stroke throughout a mean follow-up duration of 3.75 years. Future CVD was better predicted by using the combination of hs-CRP and baPWV than by using either one alone [26]. Our findings showed that older age, higher serum fasting glucose levels and DM, higher SBP, higher CRP, and poor renal function were associated with high PAS in hypertensive patients. According to multiple multivariate logistic regression analyses, age is an independent predictor of PAS in patients with hypertension, even after controlling for important covariates.

An expanding variety of extra-osseous biological roles and consequences have recently been reported to be associated with OC, an intriguing hormone produced by osteoblasts in bone [11]. A prior study has also demonstrated OC connection to the vascular system and its role in the onset of vascular calcification [12]. In addition to being produced by the bone, vascular smooth muscle cells that exhibit an osteoblast-like phenotype also express OC [12]. A systematic review has reported that although no conclusive association was established between OC and vascular calcification, the consistent presence of OC-positive cells was positively correlated with calcification [27]. Given that the kidneys remove circulating OC, OC clearance probably decreases in people with impaired kidney function [28]. A meta-analysis reported a significant inverse association between the levels of serum OC and CRP in the adult population [29]. However, in our study, the serum log-CRP levels were positively correlated with the OC levels in patients with hypertension. Many factors may have affected the consistent and inconsistent findings on OC levels and CRP, including different methods for measuring OC, potential confounders that were not considered, and only observational studies used in the meta-analysis [29]. It will take more investigation to completely comprehend the mechanisms underlying the association between OC and CRP. In a human study, patients with chronic kidney disease had greater arterial stiffness, as measured by AIx75, in correlation with higher levels of OC [13]. Subgroup analyses revealed that among young subjects, male patients, a history of hypertension or DM, and in the early stages of chronic kidney disease, and a higher serum OC level was associated with severe arterial stiffness but a better endothelial function as determined by the reactive hyperemia index [13]. Another clinical study showed that OC is associated with arterial stiffness in patients on hemodialysis [30]. Our previous clinical study also noted that elevated serum OC levels are associated with arterial stiffness in patients undergoing peritoneal dialysis [14]. According to our findings, the serum OC levels independently predict PAS in hypertensive individuals and positively correlate with either the left or right baPWV values.

Hypertension is a significant risk factor for CVD [1]. The baPWV test is non-invasive and is conducted using four pneumatic cuffs placed over the bilateral limbs for early detection of the high risk of CVD in humans [6,8,9,10]. There are also many factors that affect the baPWV, such as age, blood pressure, DM, smoking, chronic kidney disease, obesity, and metabolic syndrome [6]. Exercise, quitting smoking, and body weight reduction are lifestyle changes that enhance the baPWV [6]. The use of corticosteroids, vitamin K, or exercise is known to affect the amount of OC in the blood [28]. Our study demonstrated the correlation between serum OC and PAS measured by baPWV in hypertensive patients. Additional research is required to determine if PAS or OC is a meaningful indicator for evaluating the impact of therapies to lower the risk of CVD.

Our study has limitations due to its cross-sectional design and small sample size. Moreover, not measuring undercarboxylated OC is another limitation of our study. Undercarboxylated OC is considered the bioactive form of OC. Many tissues, including the brain, pancreas, skeletal muscle, testes, and adipose tissue, are believed to be subject to biological processes that are regulated by undercarboxylated OC. There is also increasing evidence that it is connected to the vascular system [28]. Further exploration through long-term prospective studies or randomized controlled trials with larger sample sizes is needed to comprehensively understand serum OC or undercarboxylated OC and PAS levels in hypertensive patients.

## 5. Conclusions

This study showed that in patients with hypertension, there was a positive correlation between serum OC levels and baPWV. A rise in serum OC level, with the exception of advanced age, is an independent predictor of PAS in hypertensive people.

## Figures and Tables

**Table 1:** Patient clinical characteristics with baPWV < 18.0 m/s or >18.0 m/s.

Items	All Participants (*n* = 120)	baPWV ≤ 18 m/s Group (*n* = 96)	baPWV > 18 m/s Group (*n* = 24)	*p* Value
Age (years)	65.55 ± 9.77	64.43 ± 9.31	70.05 ± 10.44	0.011 *
Height (cm)	162.11 ± 7.39	162.44 ± 7.64	160.79 ± 6.27	0.330
Body weight (kg)	70.32 ± 11.84	70.31 ± 12.07	70.33 ± 11.12	0.994
Body mass index (kg/m^2^)	26.68 ± 3.61	26.57 ± 3.68	27.12 ± 3.31	0.512
Systolic blood pressure (mmHg)	131.63 ± 16.49	129.88 ± 15.25	138.63 ± 19.54	0.019 *
Diastolic blood pressure (mmHg)	74.28 ± 10.34	74.24 ± 10.18	74.42 ± 11.22	0.941
Left baPWV (m/s)	15.06 ± 3.36	14.00 ± 2.67	19.28 ± 2.42	<0.001 *
Right baPWV (m/s)	15.11 ± 3.56	13.95 ± 2.71	19.77 ± 2.65	<0.001 *
Total cholesterol (mg/dL)	168.99 ± 36.14	169.39 ± 36.01	167.42 ± 37.39	0.812
Triglyceride (mg/dL)	128.50 (90.50–173.75)	125.50 (94.00–178.00)	136.50 (88.25–165.00)	0.847
HDL-C (mg/dL)	45.12 ± 12.74	45.39 ± 12.66	44.04 ± 13.28	0.646
LDL-C (mg/dL)	102.58 ± 27.60	102.72 ± 27.94	102.00 ± 26.78	0.910
Fasting glucose (mg/dL)	110.00 (96.00–137.00)	105.50 (94.25–131.75)	134.50 (114.50–180.25)	0.003 *
Blood urea nitrogen (mg/dL)	16.00 (14.00–20.00)	16.00 (13.25–19.00)	19.00 (14.25–23.75)	0.024 *
Creatinine (mg/dL)	1.12 ± 0.31	1.08 ± 0.28	1.28 ± 0.39	0.004 *
eGFR (mL/min)	67.32 ± 18.85	69.74 ± 17.65	57.64 ± 20.69	0.004 *
Total calcium (mg/dL)	9.13 ± 0.37	9.12 ± 0.36	9.14 ± 0.40	0.824
Phosphorus (mg/dL)	3.49 ± 0.51	3.52 ± 0.51	3.39 ± 0.49	0.261
Intact parathyroid hormone (pg/mL)	47.40 (34.00–65.68)	47.00 (37.68–65.68)	47.70 (38.03–72.40)	0.577
Osteocalcin (ng/mL)	1.57 ± 0.98	1.43 ± 0.87	2.13 ± 1.22	0.002 *
C-reactive protein (mg/dL)	0.21 (0.15–0.28)	0.19 (0.14–0.26)	0.27 (0.19–0.40)	0.007 *
Male, *n* (%)	82 (68.3)	29 (72.5)	53 (66.3)	0.488
Diabetes, *n* (%)	52 (43.3)	36 (37.5)	16 (66.7)	0.010 *
Coronary artery disease, *n* (%)	82 (68.3)	64 (66.7)	18 (75.0)	0.432
ACE inhibitor use, *n* (%)	38 (31.7)	31 (32.3)	7 (29.2)	0.768
ARB use, *n* (%)	59 (49.2)	44 (45.8)	15 (62.5)	0.144
β-blocker use, *n* (%)	64 (53.3)	49 (51.0)	15 (62.5)	0.314
CCB use, *n* (%)	46 (38.3)	34 (35.4)	12 (50.0)	0.189
Statin use, *n* (%)	69 (57.5)	54 (56.3)	15 (62.5)	0.580
Fibrate use, *n* (%)	40 (33.3)	32 (33.3)	8 (33.3)	1.000
Aspirin, *n* (%)	75 (62.5)	60 (62.5)	15 (62.5)	1.000
Clopidogrel, *n* (%)	30 (25.0)	22 (22.9)	6 (33.3)	0.292

After analysis by the Student’s *t*-test, values for continuous variables are displayed as mean ± standard deviation; variables that are not normally distributed are displayed as median and interquartile range; and categorical variables are presented as number (%) and analysis following analysis by the chi-square test. baPWV, brachial-ankle pulse wave velocity; HDL-C, high-density lipoprotein cholesterol; LDL-C, low-density lipoprotein cholesterol; eGFR, estimated glomerular filtration rate; ACE, angiotensin-converting enzyme; ARB, angiotensin-receptor blocker; CCB, calcium-channel blocker. * *p* < 0.05 was considered statistically significant.

**Table 2:** An examination using multivariable logistic regression of the variables associated with peripheral artery stiffness.

Variables	Odds Ratio	95% Confidence Interval	*p* Value
Osteocalcin, 1 ng/mL	1.797	1.077–3.000	0.025 *
Age, 1 year	1.076	1.004–1.153	0.037 *
Diabetes, present	2.303	0.539–9.830	0.260
C-reactive protein, 0.1 mg/dL	1.014	0.878–1.172	0.847
Systolic blood pressure, 1 mmHg	1.022	0.990–1.055	0.175
Fasting glucose, 1 mg/dL	1.006	0.993–1.020	0.374
eGFR, 1 mL/min	0.982	0.953–1.012	0.243

eGFR, estimated glomerular filtration rate. A statistically significant result in the multivariate logistic regression analysis was defined as * *p* < 0.05. (adopted factors: diabetes, age, systolic blood pressure, fasting glucose, eGFR, C-reactive protein, and osteocalcin).

**Table 3:** Spearman correlation coefficients between osteocalcin and clinical variables.

Variables	Spearman’s Coefficient of Correlation	*p* Value
Age (years)	0.140	0.127
Height (cm)	−0.075	0.415
Body weight (kg)	−0.065	0.480
Body mass index (kg/m^2^)	−0.026	0.780
Systolic blood pressure (mmHg)	0.091	0.323
Diastolic blood pressure (mmHg)	−0.162	0.077
Left baPWV (m/s)	0.336	<0.001 *
Right baPWV (m/s)	0.435	<0.001 *
Total cholesterol (mg/dL)	−0.023	0.804
Log-Triglyceride (mg/dL)	−0.005	0.958
HDL-C (mg/dL)	−0.030	0.744
LDL-C (mg/dL)	−0.041	0.655
Log-Glucose (mg/dL)	0.131	0.153
Log-BUN (mg/dL)	0.070	0.448
Creatinine (mg/dL)	0.176	0.055
eGFR (mL/min)	−0.208	0.023 *
Total calcium (mg/dL)	−0.004	0.961
Phosphorus (mg/dL)	−0.002	0.981
Log-iPTH (pg/mL)	0.120	0.193
Log-CRP (mg/dL)	0.211	0.021 *

Data of triglyceride, glucose, blood urea nitrogen, C-reactive protein, and iPTH levels showed skewed distribution and were log-transformed before analysis. baPWV, brachial-ankle pulse wave velocity; HDL-C, high-density lipoprotein cholesterol; LDL-C, low-density lipoprotein cholesterol; BUN, blood urea nitrogen; eGFR, estimated glomerular filtration rate; iPTH, intact parathyroid hormone; CRP, C-reactive protein. * *p* < 0.05 was considered statistically significant (2-tailed).

## Data Availability

Upon request, the corresponding author can provide the data utilized in this study.
